# IMPLEMENTATION AND IMPACT OF THE WHO INTEGRATED CARE FOR OLDER PEOPLE PROGRAM IN CHINA: A RANDOMIZED CONTROLLED TRIAL

**DOI:** 10.1093/geroni/igae098.1475

**Published:** 2024-12-31

**Authors:** Sophia Kang

**Affiliations:** Peking Union Medical College Hospital, Beijing, Beijing, China (People’s Republic)

## Abstract

This study evaluates the feasibility and impact of the World Health Organization’s Integrated Care for Older People (ICOPE) approach in urban and suburban communities of Chaoyang District, Beijing, China, amidst concerns about increasing health and social care burdens due to aging populations. Community-dwelling older adults screened as at-risk of functional declines were randomized into intervention (537) and control (1611) groups between September 2020 and February 2021. This study tested the implementation of a 6-month ICOPE intervention program against standard care. Propensity score matching was utilized to ensure comparability between intervention and control groups, totaling 938 participants. The results highlighted the feasibility of ICOPE, with high satisfaction rates among participants (97–99%) and providers (92–93%). All outcomes showed improvements after a 6-month intervention, with statistically significant least-squares mean differences (control-intervention) in vitality (Mini-Nutritional Assessment Short Form to measure vitality, −0.21, 95% CI, −0.40–0.02), mobility (Short Physical Performance Battery to measure mobility, −0.29, 95% CI, −0.44–0.14) and psychological health (Geriatric Depression Scale five items to measure psychological health, 0.09, 95% CI, 0.03–0.14) were observed (P < 0.05). These findings suggest that localizing WHO’s ICOPE approach is feasible and effective in regions with fragmented healthcare resources in China, demonstrating positive impacts on health outcomes and highlighting its potential for advancing policies and practices toward healthy aging.

